# Over-Eating

**Published:** 1860-10

**Authors:** 


### OVER-EATING

The great President Edwards acknowledged that almost every day of his life he had a battle and a defeat; the determination before going to his dinner that he would not eat beyond measure, and the confession after, that he had exceeded the limits of temperance and moderation. A venerated name, Amos Lawrence, was a greater coward, but a wiser man; for the latter years of his life he did not dare to go to the table, but had sent to his private room only as much as was proper for him. Many a man might add a score of years to his lifetime by rigidly pursuing such a practice while at home.

Few persons, perhaps, "over-eat" deliberately; it is generally done in haste, in inattention, miscalculation or inadvertence; but the consequences are the same, that is, an unmixed harm to the whole organization; the injury manifests itself in a great number of ways, according however to various laws, these effects lasting from one to a dozen hours, in every variety of intensity, from simple discomfort to actual torture. At first, there is general irritability or fretfulness for a short time after meals, eventually extending from one meal to another, until the whole existence is a growl or a groan, according to the active or passive nature of the culprit victim, who has not only blotted out his own life for all humane or noble purposes, but casts a blur and a blight over the existence of all those whose unhappy lot it has been to be placed under the same roof and to be seated at the same table. There are two ways of preventing and of curing these deplorable conditions, the manly and the mean; the manly, by going to the table twice a day, and nobly curbing the beastly appetite, saying: "I will eat this and so much, and no more by a single atom!" The mean or ignoble, by having "this and so much, and not an atom more" sent to a private table; the "this and so much," the quality and quantity, having been determined by the observed instincts and needs of the system; each man being a rule for himself, under the guidance of a wise physician, or of an unerring and competent judgment of his own. The failure of the cure of dyspepsia in countless instances has arisen from two causes. First, relying too much on medicine. Second, making another the rule for himself; when no two persons ever were alike in all conditions, therefore the same result could never take place in any two cases. In the successful treatment of dyspeptic disease, each man must be a rule to himself, adapting every thing to his individual needs, tastes, instincts, inclinations, temperament, station and habit of life. These suggestions are made to all who have force of character; to such their adoption in appropriate cases would be productive of the most happy results.

